# Effect of patient and partner preconceptional nutraceutical supplementation on the onset of natural pregnancy: a retrospective study

**DOI:** 10.1590/1806-9282.20260081

**Published:** 2026-07-10

**Authors:** Maria Julia Bauer, Hannah Halat, Peter Chedraui, Markus Lipovac, Martin Imhof, Ana Mitrovic Jovanovic

**Affiliations:** 1 Landsteiner Institute for Cell-Oriented Therapy in Gynecology – Korneuburg, Austria.; 2 Center for Gynecology and Reproductive Medicine – Vienna, Austria.; 3 Universidad Espíritu Santo, Escuela de Postgrado en Salud – Samborondón, Ecuador.; 4 Specijalna Ginekološka Bolnica Teofanović Clinic, Department of Gynecology and Reproductive Medicine – Belgrade, Serbia.

**Keywords:** Dietary supplements, Time-to-pregnancy, Reproductive health, Preconception care, Infertility

## Abstract

**BACKGROUND:**

Infertility affects 10–15% of couples worldwide. Although assisted reproductive technologies such as in vitro fertilization are effective, they are invasive, costly, and not without risks. Nutraceutical supplementation has gained importance as a non-invasive option to support natural conception.

**OBJECTIVE:**

The aim of this study was to evaluate whether combined nutraceutical supplementation improves natural pregnancy rates in subfertile couples with a relative indication for in vitro fertilization while awaiting treatment.

**METHODS:**

This retrospective study included 213 couples followed for 6 months (119 in the supplementation group; 94 controls). In the treatment group, both partners received combined nutraceutical supplementation (PROfertil® female/male). Controls received folic acid for women only, while men received no treatment. All couples were on an in vitro fertilization waiting list without an absolute indication for immediate assisted reproductive technologies. Those with oligoasthenozoospermia II/III or bilateral tubal occlusion were excluded. The primary endpoint was a natural pregnancy within 6 months.

**RESULTS:**

Natural conception occurred more often in the supplementation group than in controls (66.4 vs. 39.4%, p<0.001). Multivariate logistic regression showed a 4.13-fold higher odds of pregnancy with supplementation (95%CI 2.12–8.04, p<0.001).

**CONCLUSION:**

Combined nutraceutical supplementation significantly increased natural pregnancy rates in couples awaiting in vitro fertilization. Randomized trials are needed to confirm these findings and assess live birth outcomes.

## INTRODUCTION

Infertility affects roughly 15% of couples worldwide. Although assisted reproductive technologies (ART) have advanced, treatment success remains suboptimal, prompting interest in non-invasive adjuncts such as lifestyle optimization and nutraceutical supplementation^1^. Preconception supplementation has been linked to higher pregnancy and live birth rates^2,3^, and nutrient-rich dietary patterns correlate with increased likelihood of live birth^4^.

While individual micronutrients—including folic acid^5^, vitamin D, zinc^6^, selenium, and omega-3 fatty acids^7^—have been associated with fertility benefits, evidence on combined formulations in infertile or subfertile couples is still limited. The effect of multi-nutrient supplementation on time to pregnancy requires further investigation. Micronutrients may influence ovarian reserve markers, ovulation, endometrial receptivity, and luteal phase adequacy^8^, positioning nutritional support as a potentially accessible and cost-effective approach to enhance natural fertility and ART outcomes.

Randomized trials highlight these benefits. Nouri et al.^3^ reported that women receiving a multi-micronutrient supplement during ART produced more good-quality embryos (58%) than those using folic acid alone (36%), with a trend toward improved pregnancy rates. Similarly, adherence to a pro-fertility diet—rich in folate, vitamins B_12_ and D, fruits and vegetables with low pesticide residues, whole grains, high-fat dairy, seafood, and soy—has been associated with higher live birth rates after ART^9^ and reduced infertility risk^7^. B vitamins regulate homocysteine metabolism, and deficiencies may elevate inflammation and impair ovulation^7^. Additionally, Mediterranean-style patterns improve ART success, reduce insulin resistance, and lower ovulatory infertility risk^1^. Conversely, Western diets high in refined carbohydrates and trans fats promote inflammation and adversely affect oocyte and embryo quality^5,7^, and in men reduce sperm morphology and concentration^10^.

Several micronutrients support reproductive physiology more broadly. Omega-3 fatty acids enhance steroidogenesis, reduce inflammation, improve semen quality, support oocyte development^7^, and may increase pregnancy rates and reproductive lifespan^7,11^. In a short-term intervention, Kermack et al.^12^ found that omega-3 and vitamin D supplementation improved follicular fluid composition by increasing Eicosapentaenoic Acid/Docosahexaenoic Acid (EPA/DHA) and reducing omega-6 levels. Deficiencies in selenium, zinc, or copper have been linked to delayed conception and greater infertility risk^7^. Conditions characterized by oxidative stress—such as endometriosis and polycystic ovary syndrome (PCOS)—can impair oocyte quality^7^. Although evidence is limited, a Cochrane review suggested potential benefits of antioxidant supplementation, and coenzyme Q_10_ may improve oocyte and embryo quality^13^.

Folic acid supports Deoxyribonucleic Acid (DNA) synthesis and luteal progesterone production, reducing anovulatory cycles^5^. In men, folate deficiency increases sperm DNA damage, and combined folate–zinc therapy may enhance semen quality^14^. Additional compounds—such as glycyrrhizin—have shown reproductive benefits in PCOS models^15^.

Few studies have evaluated simultaneous micronutrient intake in couples. Veselinović et al.^16^ found that semen quality and serum micronutrient levels correlated with ART success. However, Arhin et al.^2^ highlighted inconsistent evidence for antioxidant supplementation.

A combined nutraceutical strategy may address oxidative stress, homocysteine metabolism, and hormonal balance through omega-3 fatty acids, coenzyme Q_10_, vitamin E, folic acid, selenium, catechins, glycyrrhizin, L-carnitine, L-arginine, zinc, and glutathione. These nutrients have been linked to enhanced embryo quality^3^, greater endometrial thickness^8^, and improved semen parameters^17^. This retrospective study evaluated whether simultaneous supplementation in both partners could improve fertility outcomes and shorten the time to conception.

## METHODS

### Study design and setting

This retrospective cohort study was conducted at the SGB Teofanović (Belgrade, Serbia) and the IMI Fertility Clinic (Vienna, Austria). Medical records from 213 sub-fertile couples treated between January 1, 2021, and January 31, 2022, were reviewed to compare outcomes between patients receiving nutraceutical supplementation and those receiving standard folic acid. Ethical approval was granted by the SGB Teofanović Ethics Committee (10/03/2025, EC-Nr. 49). Consent was waived due to the retrospective design, and all data were codified, anonymized, and securely stored.

### Study population

In the therapy group (n=119), both partners received the nutraceutical supplement; in the control group (n=94), women received folic acid 400 μg/day. Eligible women were aged 18–40, attempting natural conception while awaiting IVF treatment.

### Eligibility criteria

Women aged 18–40 who were trying to conceive naturally while awaiting treatment at an IVF institute were eligible if they had unexplained subfertility or fertility-affecting conditions such as PCOS. Couples were excluded if medical records were incomplete, if the woman had premature ovarian insufficiency or bilateral tubal occlusion, if the male partner had severe infertility (e.g., azoospermia), or if either partner was taking other micronutrient supplements.

### Parameters collected

#### Female

Age, body mass index (BMI), parity, gravidity, duration of time to conceive, menstrual regularity, ovulation status or stimulation (including type), tubal patency, nicotine/alcohol consumption, medication, comorbidities, baseline anti-müllerian hormone (AMH), endometrial thickness before and after supplementation, and pregnancy onset (months 1–6).

#### Partner

Age, BMI, nicotine status, urological/fertility history, and semen analysis.

### Outcomes

#### Primary

Clinical pregnancy confirmed by serum β-human chorionic gonadotropin and fetal cardiac activity. After confirmation, women transitioned to standard pregnancy supplements.

#### Secondary

Change in endometrial thickness (measured between cycle days 12–15 in the late follicular phase) before and after supplementation.

### Nutraceutical supplementation

Women in the therapy group received PROfertil Female^®^, consisting of one soft capsule with 500 mg omega-3 fatty acids and one tablet containing 30 mg vitamin E, 30 mg coenzyme Q_10_, 800 μg folic acid, 70 μg selenium, 4 mg catechins, and 12 mg glycyrrhizin. Male partners received a formulation containing two capsules with 440 mg L-carnitine, 250 mg L-arginine, 15 mg coenzyme Q_10_, 120 mg vitamin E, 40 mg zinc, 800 μg folic acid, 80 mg glutathione, and 600 μg selenium (PROfertil Male^®^). Supplements were taken daily for 6 months or until pregnancy.

### Data collection and statistical analysis

Data were extracted by an independent reviewer (Dr. Johannes Barta) and analyzed using International Business Machines Statistical Package for the Social Sciences Statistics 30.0. Continuous variables were summarized as mean±standard deviation and ranges; categorical variables as absolute and relative frequencies. Normality was assessed using histograms, Q–Q plots, and the Kolmogorov-Smirnov test. Multivariate logistic regression evaluated the association between supplementation and pregnancy, adjusting for female age, BMI, smoking, alcohol intake, ovulation stimulation, AMH, and semen quality. Changes in endometrial thickness were analyzed with an independent samples t-test. Statistical significance was set at p<0.05.

## RESULTS

Pregnancy onset was evaluated in 213 women: 119 received the nutraceutical supplement, and 94 took folic acid 400 μg/day. All therapy-group participants adhered to the protocol. Ovulation stimulation (Clomiphene or Letrozole) was used in 21% of the therapy group and 26.6% of controls. Therapy patients were treated at SGB Teofanović (56.3%) and the IMI Clinic (43.7%), while all controls were treated at IMI.

Mean female age was 35.1±4 years (range 27–43) in the therapy group and 34.4±4 years (26–42) in controls, with BMI 24.5±2.5 versus 24.2±2.4. Previous pregnancies occurred in 27.7% of therapy patients (12.6% live births) and 30.9% of controls (13.8% live births). The average infertility duration was 12±6.8 months in both groups. Cycle regularity was reported in 69.7% of therapy patients (26.9% normal ovulation) versus 66% of controls (26.6%). Smoking and alcohol consumption were similar. Baseline endometrial thickness did not differ; after 6 months, supplemented women had significantly greater endometrial thickness (10.3±1.6 mm) than controls (9.5±1.6 mm; 95%CI −1.24 to −0.37) (Table 1).

**Table 1:** Basal demographics of the therapy and control groups.

	Supplement group n=119	Control group n=94	p-value (two-sided)
Age (years)	35.1±4.0 [27–43]	34.4±3.9 [26–42]	0.190
Body mass index (kg/m^2^)	24.5±2.5 [19–29.8]	24.2±2.4 [18.8–29.8]	0.389
Gravidity (previous pregnancies)	33 (27.7)	29 (30.9)	0.619
Parity (live births)	15 (12.6)	13 (13.8)	0.793
Length of unfulfilled desire for a child (months)	11.8±6.8 [3–36]	11.8±6.8 [2–35]	0.992
Regular cycle	83 (69.7)	62 (66)	0.556
Normal ovulation	32 (26.9)	25 (26.6)	0.961
Ovulation stimulation	25 (21)	25 (26.6)	0.339
Tubal patency (one tube blocked)	51 (42.9)	59 (62.8)	0.004
Nicotine	69 (58)	54 (57.4)	0.937
Alcohol	37 (31.1)	30 (31.9)	0.898
Medication	29 (24.4)	23 (24.5)	0.987
Disease	32 (26.9)	22 (22.3)	0.561
Serum AMH (ng/mL)	2.6±1.0 [0.6–7.2]	2.6±1.0 [0.5–7.1]	0.975
Endometrium thickness before (mm) measured between cycle days 12–15	9.1±1.6 [5.0–14.1]	9.4±1.6 [6.3–13.9]	0.230
Endometrium thickness after (mm) (cycle days 12–15)	10.3±1.6 [7.5–14]	9.5 mm±1.6 [4.9–13.9]	<0.001
Partner age (years)	35.9±4.6 [22–48]	35.69±4.28 [24–47]	0.714
Body mass index (partner)	26.4±2.1 [20.6–33.8]	26.4±1.3 [25.4–32.8]	0.935
Smoking (partner)	83 (69.7)	57 (60.6)	0.164
Partner treated with proposed supplementation	119 (100)	0 (0)	<0.001
Urological fertility-related issues	10 (8.4)	7 (7.4)	0.798
Pathological semen analysis	35 (29.4)	31 (33.0)	0.694

Data is presented as mean±standard deviations (SD), ranges [minimum–maximum value], or frequencies n (%). AMH: anti-müllerian hormone.

Comorbidities occurred in 26.9% of therapy patients and 22.3% of controls, including depression, type 2 diabetes, Hashimoto’s disease, insulin resistance, hypothyroidism, microadenoma, or PCOS; 24% of both groups used medications such as antidepressants, cabergoline, levothyroxine, inositol, or metformin. Fallopian tube obstruction was observed in 42.9% of therapy patients versus 62.8% of controls. Baseline AMH averaged 2.6±1 ng/mL in both groups. Endometrial thickness increased from 9.1±1.6 mm to 10.3±1.6 mm in therapy patients and from 9.4±1.6 mm to 9.5±1.6 mm in controls.

Partner characteristics were comparable: therapy partners averaged 35.9±4.6 years and had a BMI of 26.4±2.1; 69.7% smoked, and 29.4% had abnormal semen analyses. Controls averaged 35.7±4.3 years, with a BMI of 26.4±1.3; 60.6% smoked, and 33% had abnormal semen analyses.

Pregnancy within 6 months occurred in 66.4% of supplemented couples (monthly rates: 6.7, 16.8, 9.2, 18.5, 6.7, and 8.4%) versus 39.4% of controls (6.4, 6.4, 8.5, 9.6, 2.1, and 7.4%). One control patient conceived twice (months 1 and 3) (Table 2).

**Table 2:** Monthly pregnancy rates and cumulative at 6 months among the studied groups.

Studied group	1st month	2nd month	3rd month	4th month	5th month	6th month	Total
Supplemented	8 (6.7)	20 (16.8)	11 (9.2)	22 (18.5)	8 (6.7%)	10 (8.4)	79 (66.4)
Control	6 (6.4)	6 (6.4)	8 (8.5)	9 (9.6)	2 (2.1)	7 (7.4)	37 (39.4)
p-value	0.921	0.021	0.852	0.067	0.115	0.798	<0.001

Multivariate logistic regression, adjusted for female age, BMI, smoking, alcohol, ovulation stimulation, AMH, and semen analysis, showed a significant model (*χ* ^2^ [11, n=213]=44.14, p<0.001) with good fit (Hosmer-Lemeshow *χ* ^2^ [8]=8.27, p=0.408), explaining 25% of variance (Nagelkerke R^2^=0.25) and correctly classifying 73.2% of cases. Nutraceutical supplementation increased the odds of pregnancy 4.13-fold (95%CI 2.12–8.04, p<0.001). Maternal age (OR 0.87, p=0.002) and BMI (OR 0.86, p=0.02) decreased pregnancy likelihood, while higher AMH increased it (OR 1.45, p=0.043).

## DISCUSSION

This study found that targeted nutraceutical supplementation significantly improved natural conception, with 66.4% of women achieving pregnancy within six months versus 39.4% of controls. These findings support prior evidence of improved conception rates with similar preparations^18^, highlighting the potential of nutraceutical strategies in fertility care.

Supplementation also benefits semen quality. L-carnitine and vitamin E enhance sperm motility, concentration, and morphology, while L-carnitine counteracts reactive oxygen species. Long-term coenzyme Q_10_ use improves progressive motility^19^. Selenium mitigates oxidative stress, supports DNA repair, and improves semen parameters^20^. Combined male supplementation with L-carnitine, L-arginine, coenzyme Q_10_, zinc, glutathione, selenium, and vitamins C and B_9_ increases total and progressive motility, reduces DNA fragmentation, and enhances pregnancy rates^21^.

ART studies show higher rates of good-quality embryos and trends toward improved pregnancy with female nutraceutical supplementation^3^; male-partner antioxidant use also improves implantation and clinical pregnancy^22^. Adherence to pro-fertility or Mediterranean diets correlates with better ART outcomes and live births^23^, while in men, Western-style diets promote inflammation, oxidative stress, hormonal imbalance, and impaired semen parameters^10^.

Mechanistically, folic acid and vitamins B_6_ and B_12_ regulate homocysteine metabolism; elevated homocysteine impairs ovulation, embryo quality, and increases miscarriage risk^24^. Omega-3 fatty acids and coenzyme Q_10_ support steroidogenesis, oocyte quality, and reduce oxidative stress^25^. The evaluated supplement likely acts synergistically via these pathways.

The proposed intervention is non-invasive, low-risk, and inexpensive, offering a safe adjunct to ART or natural conception. However, standardized recommendations for women attempting conception remain needed. Strengths of the study include standardized supplementation for both partners and clinically relevant outcomes. Limitations involve modest sample size, lack of dietary control beyond supplementation, reliance on self-reported adherence, absence of mechanistic biomarkers beyond AMH, and unavailable live birth data.

To our knowledge, few studies have assessed simultaneous supplementation in both partners and its impact on natural conception. Large multicenter randomized trials are needed to identify mechanisms and responsive subgroups and explore combined male-female supplementation.

In conclusion, nutraceutical supplementation may enhance fertility potential in both partners by modulating homocysteine metabolism, balancing oxidative stress, and improving semen quality and hormonal regulation. Clinically, it represents a safe, low-cost, non-invasive adjunct to natural conception and ART.

## Data Availability

The datasets generated and/or analyzed during the current study are available from the corresponding author upon reasonable request.
